# Markers of endothelial dysfunction in patients with severe sepsis resuscitated with hydroxyethyl starch 130/0.42 vs. ringer's acetate

**DOI:** 10.1186/2197-425X-3-S1-A793

**Published:** 2015-10-01

**Authors:** RB Müller, SR Ostrowski, N Haase, J Wetterslev, A Perner, PI Johansson

**Affiliations:** Rigshospitalet, Dept. of Intensive Care, 4131 Copenhagen, Denmark; Rigshospitalet, Section for Transfusion Medicine, Copenhagen, Denmark; Rigshospitalet, Copenhagen Trial Unit, Copenhagen, Denmark

## Introduction

The Scandinavian Starch for Severe Sepsis/Septic Shock (6S) trial [1] showed increased mortality in patients with severe sepsis resuscitated with hydroxyethyl starch 130/0.42 (HES) vs. Ringer's acetate. The underlying pathophysiological mechanisms are, however, not fully elucidated. The endothelium may be important for the effects of fluid therapy, and the different fluids may impact the endothelium differently.

## Objectives

We aimed to investigate associations between HES vs. Ringer's and plasma biomarkers reflecting endothelial damage and function, including its natural anticoagulant systems, and if they were associated with 90-day mortality. We hypothesized that allocation to HES increased endothelial dysfunction and hence mortality.

## Methods

Six biomarkers: soluble trombomodulin (sTM); syndecan-1; soluble CD40 ligand (sCD40L); plasminogen activator inhibitor-1 (PAI-1); tissue-type plasminogen activator (tPA) and protein C were assessed in a subgroup of 208 patients from the 6S trial [1]. We analyzed differences in plasma concentration from baseline to day 2 after randomisation in the two intervention groups using linear or logistic regression models. Multiple imputations were used for missing data and the analyses were adjusted for stratification variables (university hospital, shock and hematologic malignancy) and baseline SAPS II and use of plasma.

## Results

At baseline the HES vs. Ringer's groups were comparable regarding age (median 66 vs. 66, p = 0.69), disease severity (SAPS II (52 vs. 55, p = 0.73)) and type of admission (surgical 45% vs. 39%, p= 0.35). Also, the baseline concentrations of the biomarkers did not differ between the intervention groups. The masked trial fluid was administered in comparable volumes in the HES vs. Ringer's groups in the first 2 days (2250 ml vs. 2500 ml; p = 0.73). In both adjusted and unadjusted analyses the Ringer's group had a higher increase in plasma sTM (baseline to day 2) as compared to the HES group (1.78 ng/ml; 95% confidence interval (CI) 2.88 to 0.67; p = 0.002). However, sTM was not associated with mortality; increase in PAI-1 was the only biomarker change associated with 90-day mortality (odds ratio for 1 unit increase was 1.04; 95 % CI 1.01-1.08; p = 0.01).

## Conclusions

We could not confirm that HES increased endothelial dysfunction in this population. On the contrary, the Ringer's group had higher circulating sTM concentrations on day 2 indicating increased endothelial damage. Thus, the increased mortality observed with HES in patients with severe sepsis may not be explained by endothelial dysfunction.

## Grant Acknowledgment

The 6S trial was funded by the Danish Research Councils and Rigshospitalet and supported by the ACTA foundation and B Braun Medical. We warmly thank all the members of the 6S trial group.
